# The Association of Job and Family Resources and Demands with Life Satisfaction through Work–Family Balance: A Longitudinal Study among Italian Schoolteachers during the COVID-19 Pandemic

**DOI:** 10.3390/bs11100136

**Published:** 2021-10-06

**Authors:** Alfonso Landolfi, Massimiliano Barattucci, Assunta De Rosa, Alessandro Lo Presti

**Affiliations:** 1 Dipartimento di Psicologia, Università Degli Studi Della Campania “Luigi Vanvitelli”, Viale Ellittico, 81100 Caserta, Italy; alfonso.landolfi@unicampania.it (A.L.); susi_derosa@yahoo.it (A.D.R.); 2 Faculty of Psychology, e-Campus University, 22060 Novedrate, Italy; massimiliano.barattucci@uniecampus.it

**Keywords:** work–family balance, life satisfaction, job control, supervisor support, family support

## Abstract

Successfully balancing between work and family domains represents a major issue to both employees and employers, especially during COVID-19 pandemic times during which employees are often forced to work from a distance and turn to home-schooling. An occupational group particularly affected by work changes due to COVID-19 pandemic restrictions is represented by schoolteachers. We aimed at examining the associations between some job-related and family-related antecedents on the one hand and, on the other, life satisfaction as an outcome, including work–family balance as a mediator. A total of 357 Italian teachers completed a questionnaire at two different times: job control, coworkers support, supervisor support, workload, family support, and family workload were assessed at Time 1; and work–family balance and life satisfaction were assessed at Time 2. Both data collections were performed during the COVID-19 pandemic. The hypothesized direct and indirect relationships were tested by utilizing structural equation modeling. Significant and positive indirect effects of focal predictors towards life satisfaction through work–family balance were found for job control, supervisor support, and family support. The paper contributed to the literature by testing Grzywacz and Carlson’s theoretical conceptualization of work–family balance and by attempting to delineate its repertoire of potential antecedents among schoolteachers. From a practical point of view, the present study emphasizes the crucial role that certain job antecedents and family antecedents play in promoting teachers’ work–family balance and life satisfaction.

## 1. Introduction

The work–family interface is potentially an important issue for every worker, including those working in the educational sector [1]. A few decades ago, the US Department of Labor [2] suggested that a successful balance between work and family domains would become a major issue relative to the workforce and for organizations in attracting and retaining high-potential workers. Social and economic changes intervened in the last decades; in particular, the increasing ratio of dual-earning couples has gradually opened up spaces for a renewed focus on work–family balance [3]. Additionally, the COVID-19 pandemic, which, from early 2020, induced worldwide diffusion of a new respiratory virus and increase in victims’ chances of hospitalization and death (with consequent effects on the healthcare systems in terms of care prioritization to the disadvantage of other pathologies), had a strong impact on families, workers, and their organizations. In particular, families were challenged to find new methods of balancing between parental and work roles in light of the closure of educational settings (e.g., schools), the increased use of remote work from home and the resulting higher chances of work-to-family (e.g., overtime work) and family-to-work (e.g., children’s needs during working hours) conflicts, in addition to decreased investment in organizations with respect to family friendly initiatives such as employees’ kindergartens and parental leaves.

In this scenario, work–family balance (henceforth, WFB), defined as the “accomplishment of role-related expectations that are negotiated and shared between an individual and his or her role related partners in the work and family domains” [4], is emerging as a more holistic concept than compared to work–family conflict and work–family enrichment [5,6]. Taking a balanced perspective means not contrasting work and family domains, nor identifying an originating domain with respect to a receiving domain (e.g., work-to-family vs. family-to-work). Instead, it involves encompassing both family and work domains and both dimensions simultaneously: conflict and enrichment [7]. In fact, WFB can be conceptualized as distinct from conflict and enrichment [8], with the former defined as “a form of inter-role conflict in which the role pressures from the work and family domains are mutually incompatible in some respect” [9] (p. 77) and the latter defined “as the extent to which experiences in one role improve the quality of life in the other” [10] (p. 73).

As for WFB antecedents, several authors have found evidence of the predictive value of social support in particular (e.g., coworkers support, supervisor support, and family support), as well as of family-supportive organizational culture [8,11], underlining the social, dynamic, and interactive nature of psychosocial phenomena that may result in increased WFB. As for WFB outcomes, different studies suggest that WFB has beneficial consequences on all life domains [12], being negatively associated with anxiety and depression [13] and positively related to family and job satisfaction [14]. Moreover, WFB has important implications on people’s wellbeing and work productivity [15], and it also relates to family performance and organizational commitment [16]. However, while the evidence with respect to WFB antecedents [7,17] and outcomes [13,16] is well established, less scholarly attention has been devoted to the examination of WFB as a mediator between work-antecedents and family-antecedents and individual outcomes, especially those focusing on a peculiar occupational group of teachers (see next section for details).

From a theoretical standpoint, Grywacz and Carlson [4] emphasized the social dimension of WFB by focusing on the fulfilment of responsibilities related to both work and family roles and by suggesting that the positive and negative experiences that each individual experiences, both in work and family domains, shape their perception of balance; thus, the evaluation of individual and contextual antecedents is compelling for understanding the factors that foster or hinder WFB. In this study, consistently with Grzywacz and Carlson [4], we argue that it is essential to accurately characterize work–family balance in order to focus on the intrinsic interactional aspects of daily work and family life: In this vein, the authors underlined that a fundamental contribution to the individual experiences is provided by the entire set of formal and informal relationships with colleagues and supervisors (as well as own family members), which impacts life satisfaction and family-related and job-related perceptions. In particular, the increasing importance of social relationships at work has recently been stressed by Horan and colleagues [18], who introduced the concept of personal workplace relationships (refer the rest of the paper for a detailed description) and emphasized several aspects that not only characterize social relationships within work contexts, but also all their beneficial effects for the individual at work as well as their significant alternative domains (e.g., family and community) and related actors (e.g., partners and friends).

Building consistently with Grywacz and Carlson on this scenario [4], we examined the associations between some job-related (i.e., job control, coworkers support, supervisor support, and workload) and family-related (i.e., family support and family workload) antecedents on the one hand, and life satisfaction on the other, including WFB as a mediating variable, on a sample of Italian school teachers sampled during the 2020 COVID-19 pandemic. In conducting this examination, this study contributes to the literature by shedding more light on the role of WFB as a mediator on the differential predictive role of several job-related and family-related antecedents with respect to life satisfaction, particularly with reference to an occupational group that has usually been overlooked in work–family studies [1], i.e., schoolteachers.

In the next sections, we will introduce the work–family interface, as well the more general occupational issues and peculiarities of teachers as an occupational group, outline our theoretical bases, and advance our study hypotheses in the light of previous studies and shortfalls in the literature.

## 2. Italian Schoolteachers

In the Italian context, working as a primary and secondary education teacher currently requires the achievement of both a degree and further post-graduate specialization courses which make the profession very stimulating and demanding. Therefore, nowadays, this profession also requires frequent updates and commitments that must be reconciled with family needs.

Within the realm of work–family interface studies, for different reasons, researchers did not perceive teachers as a fruitful target for their studies [19]. Only a few studies have dealt with analyzing the work–family interface of teachers [20], with most of the research focusing on their work–family conflict [19,21] and enrichment [22].

Today, however, also due to the various and dramatic cultural, social, and organizational changes that are also affecting the educational sector and those working in it, the need to pay attention to the problems associated with the necessity to balance different roles has grown among teachers. In the wake of the above, WFB has eventually become an essential issue for all occupational categories, including those employed in teaching positions.

Therefore, the success of balancing life domains also represents a major challenge for teachers [23]. In fact, besides strict competence, teaching passionately, thus devoting time and energy to students, will more effectively contribute to the functioning of the educational system, especially in the COVID-19 pandemic scenario characterized by frequent and recurring distance learning and home-schooling. The educational sector faces several challenges to meet global needs and expectations [24]: teachers are required to be constantly committed to ensuring that educational programs will be not only effective and efficient, but also helpful for students’ growth as individuals and citizens [25].

Reconciling work and family and being satisfied with one’s life thus remain a compelling challenge to teachers [1]. The concept of satisfaction linked to different life domains has been commonly conceptualized as life satisfaction, defined as a global assessment of a person’s quality of life according to their chosen criteria [26], and has been extensively studied in the educational sector [27].

Furthermore, another fundamental aspect that we aim to highlight in this study concerns the effects of the COVID-19 emergency. The consequences of the recent pandemic have significantly transformed the work environment and job demands, especially for teachers, mostly women, often with high family burdens, which has seriously threatened professional satisfaction and job quality [28,29].

Due to the COVID-19 pandemic, schools and training institutes have been made physically inaccessible as a preventive strategy; teaching activities were provided at a distance, exposing students, teachers, and their loved ones to new work and family scenarios, often merged with each other. Teachers faced all the practical aspects of the transition to distance learning: the transformation of contents, time management, management of different technologies and digital tools, sharing of workspaces, and management of the online classroom, etc.

This change in their work setting has led to heightened overload and work stress, mainly due to unsuitable work conditions, organizational factors within the family (e.g., working times, learning times of children, working times of cohabitants, and working times of other family members, etc.), and the need to re-plan and re-organize work and family activities [30,31].

Several studies have examined the psychological effects in the context of substantial overload and overlapping of demands between work and family, which characterized the different phases of lockdown and reopening [32]. In this study, we set ourselves the goal of analyzing both the contribution of work and family resources to WFB and that of work and family demands which, in this specific situation, can represent—in turn–the additional requests to schoolteachers.

## 3. Theoretical Framework

Several studies have shown that the simultaneous participation in family and work roles generates stress in an individual’s life [8] and these conflicting situations may also negatively affect the person’s health and well-being [32]. Managing the boundaries of work and family roles is important for a person’s life [33] and the lack of balance between work and family roles can lead to negative outcomes.

According to Grzywacz and Carlson, WFB focuses on general relationships that accompany a role (e.g., important others at home) rather than specific relationships (e.g., spouse) [4]. This emphasis on social relationships calls again into cause the concept of personal workplace relationships, especially in the wake of Clark’s [34] work/family border theory. According to this theory, individuals can be seen as perpetual border-crossers across both their family and work domains, engaging in segmentation and, especially for the purpose of our study, integration activities of these two main domains. Consistently with these assumptions, personal workplace relationships, but also more general personal relationships, can be meant as a form of work/life blending [18]: satisfactory social relationships at work spill into the home domain (e.g., granting additional resources for family chores) and satisfactory relationships at home spill into the work domain (e.g., releasing additional energies for unexpected overtime work). WFB can be seen as an individual global perception at the intersection of these two domains and the personal and formal relationships that take place into them: a higher WFB will imply being able to successfully negotiate and accomplish expectations at work and home from one’s own significant others (e.g., spouse, supervisor), thus developing and nurturing satisfactory personal relationships in both domains, while a lower WFB will mean not being able to balance between work and family, hence entering a vicious circle of negative spillover and crossover effects (e.g., strain, social undermining) that will affect the individual’s relationship and ultimately, their quality of life either at home or at work or both.

Thus, it appears that WFB has important implications for people’s well-being and work productivity [15]. Moreover, with regard to WFB-related effects, it has been shown that individuals who perceive a balance between the family and work domains tend to be more satisfied with one’s own life and report better physical and mental health [11,16,35]. Therefore, in an attempt to examine how WFB can mediate the effects of the specific job and family characteristics on teachers’ life satisfaction, the decision to adopt Grzywacz and Carlson’s WFB model [4] as a framework was implemented. In particular, it is expected to found WFB to mediate the associations between job- and family-related demands and resources and life satisfaction, as successfully balancing between work and family (regardless of the fact of marital status, of having children, pets, hobbies, community roles, etc.) domains is fundamental to virtually any individual and thus can have a great impact on their life satisfaction [36]. At the same time, job- and family-related demands and resources may significantly impact WFB (as we will detail in the next two sections) by releasing or depleting energies and resources that could be invested in both domains, ultimately resulting in higher or lower levels of life satisfaction [37,38] through their previous impact on the individual’s work–family interface quality (i.e., WFB). In fact, with particular reference to this last issue, Grzywacz and Carlson [4] suggested that the positive and negative experiences that each individual experiences shape their perception of balance. Therefore, it is compelling to examine these antecedents to be able to design effective interventions. Certain job and family characteristics may influence the work–family interface [16,39,40] and therefore the experience of balance between the roles played by workers within the work and family domains.

## 4. Work-Related Resources and Demands

Job demands and resources play an important role as antecedents in the relationship between work and family domains [8]. On one side, job demands (i.e., workload) can lead to a deterioration of both physiological and psychological health as they require a psycho-physical effort; on the other side, job resources (i.e., job control, coworkers, and supervisor support) facilitate the achievement of work objectives and stimulate growth, learning, and personal development [41]. Among the different resources and demands, workload, job control, coworkers, and supervisor support traditionally played an important role as antecedents of WFC and WFE [42,43]. Therefore, being conflict and enrichment two complementary constructs to WFB, it is worth examining job resources and demands as antecedents of WFB. In particular, we can expect that job resources will contribute to increasing WFB as they will release or make available resources (e.g., time, skills, advice, and motivation) that the individual will use during their work activity, resulting in heightened performance, time and energy savings, increased expertise, and subsequent beneficial effects on their ability to balance between work and family. For instance, coming back from work less tired as the individual enjoyed adequate supervisor support, the opportunity to control their work pace, etc. may save energies that they could fruitfully invest in taking care of their family (e.g., playing with children, helping the spouse with family chores, or enjoying a hobby). Conversely, job demands will result in lowered WFB, as they imply the loss of additional resources (e.g., time, energies) that the individual would usually invest in their family activities. Hence, for instance, working overtime will mean having less time for enjoying one’s relationship with their spouse, or experiencing a higher workload will result in stress and fatigue that will not make the individual able to cope adequately with family needs and demands (e.g., do grocery shopping before coming home from work).

Available evidence suggests that employees with higher job control have fewer family-to-work conflicts and experience less stress [44] as they can more easily manage their job in the face of potential work–family interferences. Employees tend to feel more positive when they perceive greater control over their work environments. For example, the psychological experience of job control helps to reduce stress and is associated with feelings of well-being and fulfillment [45]. Grzywacz and Marks [46] found that control was indeed linked to positive work–family spillover. Hence, it follows that employees who have higher job control may be more able to combine their work and family lives, and ultimately be more satisfied with their overall life. 

Bellavia and Frone [47] found that support provided by supervisors and coworkers negatively impacted the levels of conflict between the work and family domain. Moreover, social support, coworkers’ support, and workload seem to be fundamental antecedent variables in the work–family context [3,48]. Support provided by both supervisors and coworkers is amongst the most studied job resources [49]. Coworkers’ support can be defined as the perceived support given by colleagues [49]; instead, supervisor support is meant as the support provided by supervisors to stimulate through feedback, appreciation, and contributing to employees’ work [50]. It must be noted that coworkers and supervisor support do not necessarily imply only formal and organizational role-based relationships, but also personal workplace relationships that can prove fundamental in promoting the individual’s WFB. In fact, enjoying a supportive relationship that is voluntary with coworkers and supervisors that they get to know well, which is characterized by a strong and positive emotional component, and is mutual and consensual, can have an added value that goes well beyond technical and informative support.

However, while several studies have examined the role of job resources and job demands on stress, there is less evidence concerning the impact of these resources on life satisfaction. Additionally, few studies have examined the role played by the workload on life satisfaction [51]. The term “workload” refers to “all activities including employees’ time spent in performing professional duties responsibilities and interests at work, either directly and indirectly” [24]. Nevertheless, despite the urge to track the impact of job demands such as workload on individuals’ outcomes in the labor market, there is limited evidence on the relationship between workload and life satisfaction [52]. The existing findings suggest that workload is a stressor and has a long-term negative impact on well-being [53] as well as work–family interface [54]. Some studies argue that when the workload is higher, it causes greater vulnerability to stress by reducing life satisfaction [55]. Thus, the following was predicted:

**H1.**  *The positive associations between job control (a), coworkers support (b), and supervisor support (c) with life satisfaction will be mediated by WFB.*

**H2.**  *The negative association between workload and life satisfaction will be mediated by WFB.*

## 5. Family-Related Resources and Demands

Family resources and demands also play an important role in the work–family context as they can positively (e.g., family support), or negatively (e.g., family workload), interact with the work domain. Enjoying family support can be fundamental for WFB as it will allow the individual to acquire and potentially invest additional resources in the family domain (e.g., spending time with children while the spouse will take care of housecleaning) and/or the work domain (e.g., being able to leave for a business trip as the spouse will take care of the family). Conversely, the higher family workload will be more likely to deplete resources that will make the individual less able to balance between work and family due to reduced resources (e.g., taking care of an ill relative may result in less time to be devoted to family and/or work activities).

In line with Hobfoll’s COR theory [56], social support can be considered a fundamental resource for workers as individuals are motivated to acquire and protect the things they value [57]. Social support has beneficial effects in reducing stress and increasing life satisfaction [58]. Scholars emphasized that “social support from family members provides workers with more resources via instrumental and affective means” [59] and is linked to an increase in well-being [60]. We can define family support as a resource for dealing with work–family challenges. While several studies have examined the impact of family on work, there is limited evidence about the mediating role that the WFB may play by linking family support and life satisfaction. Some studies suggest that family support may influence individuals’ experiences of WFB [11].

Nicklin and Mcnall [61] proved that family support drives family satisfaction via affect, and the resources that are generated are transferred to the workplace. While studies on instrumental support are contrasting, Wayne et al. [62] found that family support did not predict family–work enrichment, however, it remains an important resource for counteracting negativity in the workplace [63]. Orellana et al. [59] examined the well-being of dual-income parents and found that perceived family support was positively associated with WFB and life satisfaction for both partners. These results are consistent with studies performed on crossover and spillover effects, as through these processes the experiences of an individual influence both the various domains of life and other individuals [64]. 

Less attention has been paid to the study of family workload. Hobfoll [56] explained that individuals attempt to cope with stressful events through the use of resources, when the balance is not re-established and the individual continues to lose resources, they become more vulnerable. The environment can either hinder or favor the enrichment of individual resources, as in the case of the family environment [51]. The family workload can be defined as the excessive demands coming from the family environment. This can be caused by excessive caring responsibilities, unsupportive family members, or the presence of a baby at home [65]. Some studies emphasized the role of family–work conflict which occurs when demands in the family domain interfere with the work domain [43]. Based on this scenario, the following was hypothesized:

**H3.**  *The positive association between family support and life satisfaction will be mediated by WFB.*

**H4.**  *The negative association between family workload and life satisfaction will be mediated by WFB.*

## 6. Method

### 6.1. Participants

Three hundred and fifty-seven Italian teachers, belonging to different Italian schools, were contacted within schools by trained researchers. All participants taught in primary, secondary, or high schools. At the time of the data collection, they were in remote work mode. Regarding gender, 138 (37.7%) were men and 219 (61.3%) were women, with age ranging between 18 and 65 years (M = 44.78; SD = 12.35). Regarding the number of children, 48 participants (14.7%) had no children, while 309 participants (86.3%) had at least one child, while as for marital status, 81.6% of the respondents were married or cohabiting.

In regard to the educational level, 117 (32.8%) had a high school diploma, and 240 (67.2%) had a university degree or higher. About the employment status, 290 respondents (81.2%) had a permanent employment contract, while 64 (17.9%) had a temporary/fixed-term contract. Their average general tenure was 21.53 years (SD = 12.51).

### 6.2. Procedure

Participants were voluntarily recruited through a convenience sampling strategy.

In particular, the first author contacted several primary, secondary, and high schools a maximum of 50 km away from his university, introduced their principals to the research project and its aims, the methodology, and the questionnaire that had been developed for testing the study hypotheses, the data collection procedure, and asked for the involvement of teachers through internal communication channels (i.e., e-mails, bulletin board messages). Those who agreed received a copy of the questionnaire inside a sealable envelope and were invited to fill it in the next two weeks. As for the t2 questionnaire, teachers received an e-mail containing a Google Forms link to access the second CAWI questionnaire.

This study was planned according to both the Italian law on data protection (General Data Protection Regulation) and the Helsinki Declaration [66], and all participants provided their informed consent, participation being voluntary.

Data were collected at two different times during the COVID-19 pandemic; the first data collection was carried out in March 2020 while the second one was between June and July 2020. At Time 1, participants completed a paper-and-pencil self-report questionnaire including scales about job and family resources and demands. The instructions for participation, the objectives of the study, and a declaration on data processing in compliance with current Italian laws were reported on the first page of the questionnaire. At Time 2, after about 3 months, participants were asked to fill in a second questionnaire including scales assessing WFB and life satisfaction. The time-lagged design was used to avoid the common method bias, and in order to reduce method biases caused by commonalities in scale endpoints and anchoring effects were used different formats and scale endpoints. 

### 6.3. Measures

Job control [67]; Italian version by Lo Presti and Nonnis [68] was assessed through three items (e.g., “I can decide myself how I execute my work”; “I have sufficient autonomy in deciding how to do my job”). It refers the ability of a worker to feel able to influence what happens in their work environment, in particular to influence matters relevant to their personal goals. Participants used a five-point frequency scale ranging from 1 = never to 5 = always. Cronbach’s alpha was 0.90.

Coworker support [67]; Italian version by Lo Presti and Nonnis [68] included three items (e.g., “If necessary, I can ask for the help of my colleagues”). It refers to the perception of support received from one’s colleagues in the workplace, or rather the extent to which employees believe their colleagues are willing to assist them about their goals. Responses were collected through a five-point frequency scale (from 1 = never to 5 = always). Cronbach’s alpha was 0.90

Supervisor support [69]; Italian version by Lo Presti and Nonnis [68]: included three items (e.g., “I know I can rely on my supervisor/manager when I need it”). It refers to the perception of support received from their supervisors in the workplace, or rather the extent to which employees believe their supervisors are willing to provide them with assistance about their goals and work needs. Responses were collected through a five-point frequency scale (from 1 = never to 5 = always). Cronbach’s alpha was 0.94.

Workload [67]; Italian version by Lo Presti and Nonnis [68]: three items (e.g., “I have too much work to do”). The measure refers to the quantitative and demanding aspects perceived by the employees in relation to their work duties. Responses were collected through a five-point frequency scale (from 1 = never to 5 = always). Cronbach’s alpha was 0.82.

Family support was assessed through twelve items (e.g., “When I have a problem at work, my family members are worried about me”) from the Family Support Inventory [63,70] and refers to the perceived extent of affective and instrumental family support. It refers to the perception of the support received by their family members, i.e., the extent to which the person believes that their family members are willing to provide them with instrumental and emotional assistance about their needs. Participants responded through a five-point scale ranging from 1 = completely false to 5 = completely true. Cronbach’s alpha was 0.84.

Family workload [70] was assessed through 6 items that asked to evaluate the frequency according to which the individual was in charge to accomplish a series of family chores within own household (e.g., “Wash the dishes”, “Iron the clothes”, etc.). The measure refers to the quantitative and demanding aspects perceived by people concerning their tasks in the family domain. Responses were collected through a five-point frequency scale (from 0 = never to 4 = always). Cronbach’s alpha was 0.94.

Work–family balance [16]; Italian version by Landolfi and Lo Presti [71] assesses the extent to which a person perceives (s)he is meeting external parties’ work and family role expectations for them. It comprised 6 items, (e.g., “I am able to negotiate and accomplish what is expected of me at work and in my family”), with a five-point Likert scale from 1 = completely disagree to 5 = completely agree. Cronbach’s alpha was 0.95.

Life satisfaction [72]; Italian version by Lo Presti, Molino, Emanuel, Landolfi, and Ghislieri [73] refers to the extent to which the individual is satisfied with their own life. It was assessed via five items (e.g., “I am satisfied with my life”) with a five-point Likert scale ranging from 1 = completely disagree to 5 = completely agree. Cronbach’s alpha was 0.94.

### 6.4. Data Analysis

Missing values were replaced by the Expectation Maximization method (SPSS 21) [74].

Descriptive statistics and correlations were calculated through IBM SPSS 21 in order to investigate associations between variables. Structural equation modeling analyses (Lisrel 9.3) using the Robust Maximum Likelihood estimation method were used to evaluate the measurement and structural models concerning the study variables and their associations (along with the indicators’ covariance matrix).

Fit indices that minimized the likelihood of Type I and Type II errors [75] were selected. These indices included the chi-square test (χ^2^), the comparative fit index (CFI), the non-normed fit index (NNFI), the standardized root mean residual (SRMR), and the root mean square error of approximation (RMSEA; with 95% confidence interval lower and upper limits, hereafter 95% CI (LL, UL)). In particular, while a significant χ^2^ can indicate a poorly fitting model, as this test is affected by sample size, it is not reliable when used in larger samples. Therefore, we have added the above-mentioned alternative fit indices. Criteria for the goodness of these fit indices can range from less (CFI, NNFI ≥ 0.90; SRMR, RMSEA ≤ 0.10) to more conservative criteria (CFI, NNFI ≥ 0.95; SRMR, RMSEA ≤ 0.08).

## 7. Results

### 7.1. Descriptive Statistics

Table 1 depicts the descriptive statistics and zero-order correlations of all study variables.

A measurement model was developed in order to examine the construct validity of study measures using confirmatory factor analysis (CFA); a common method is to compare two models, a one-factor model and a complete model containing as many factors as included measures (in our case, eight latent variables). The use of the CFA aims to verify the hypotheses formulated about the structure of the relationships between variables. In this case, through the CFA we have confirmed the validity of the constructs we intend to measure and we have also evaluated how the items used are measures of the construct. The two models were compared on the basis of chi square/degrees of freedom scores, and different goodness of fit indices (Table 2).

A remarkable improvement of all goodness of fit indexes of Model 2 (eight factors) compared to Model 1 (one factor) can be observed. In more detail, Model 2 showed satisfactory goodness of fit indexes (χ^2^ = 2035.01, df = 779, CFI = 0.95, RMSEA = 0.07, SRMR = 0.11, and NNFI = 0.95) providing support for construct validity of all study variables. If the indices of Model 1 had been significant, an inadequacy of the hypothesized measurement model could have been hypothesized. 

### 7.2. Direct and Indirect Associations

**Figure 1:**
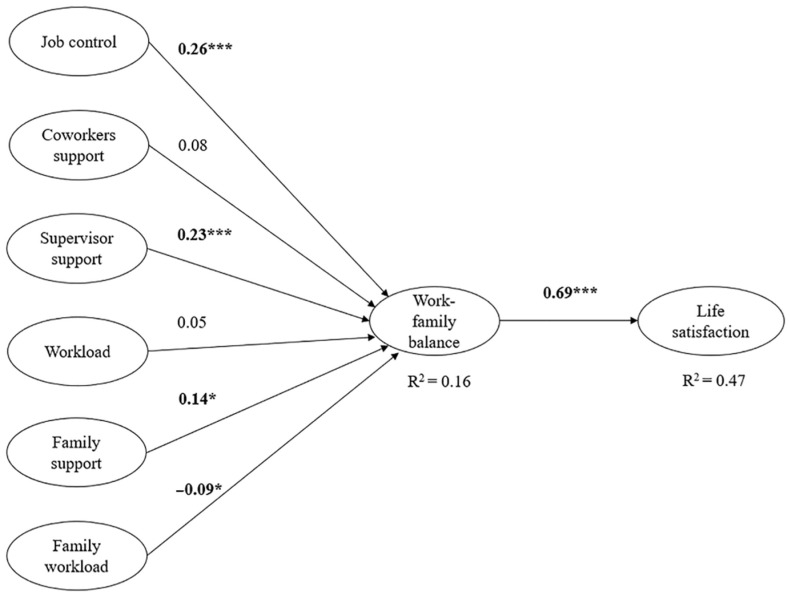
Structural model (standardized coefficients). Note: * *p* < 0.05; ** *p* < 0.01; and *** *p* < 0.001.

The hypothesized indirect relationships were tested through a structural model (Figure 1). The estimated model showed satisfactory goodness of fit indices: χ^2^ = 1739.81 df = 772, CFI = 0.95, SRMR = 0.1, RMSEA = 0.061 CI (0.058; 0.065), and NNFI = 0.95.

Hypothesis one predicted that work–family balance would mediate the positive associations between (a) job control, (b) coworker support, and (c) supervisor support with life satisfaction. Results indicate that significant and positive indirect effects of focal predictors towards life satisfaction through work–family balance were found for job control (β = 0.18, *p* < 0.001) and supervisor support (β = 0.16, *p* = 0.02). As for the direct effects, work–family balance was positively predicted by job control (β = 0.26, *p* < 0.001) and supervisor support (β = 0.23, *p* < 0.001). The relationships of coworkers’ support with work–family balance were not significant (β = 0.08). Hence, hypothesis one was partially supported.

Hypothesis two predicted that work–family balance would mediate the negative associations between workload with life satisfaction. Results showed no significant effects (β = 0.05, *p* > 0.05). Hence, hypothesis two was not supported.

Hypothesis three predicted that work–family balance would mediate the positive associations between family support and life satisfaction. Results indicate that significant and positive indirect effects of family support towards life satisfaction through work–family balance were found (β = 0.10, *p* = 0.04). Thus, hypothesis three was supported.

Hypothesis four predicted that work–family balance would mediate the negative associations between family workload and life satisfaction. Results indicate only significant direct effects: Work–family balance was negatively predicted by family workload (β = −0.09, *p* = 0.05). Hypothesis four was not supported.

As regards other direct effects, life satisfaction was positively predicted by work–family balance (β = 0.69, *p* < 0.001).

In regard to the explained outcome variables’ variance, predictors explained a significant amount of variance in work–family balance (16%, *p* < 0.001) and in life satisfaction (47%, *p* < 0.001).

Finally, our results show that work–family balance fully mediates the relationship between some family and job resources, and life satisfaction. No mediation effect of work–family balance was found between family and job demands and life satisfaction. To disentangle any potential alternative explanation about the relationships between study variables, we tested an alternative structural model containing effects from predictors to both WFB (as in the previous model) and life satisfaction (thus excluding the indirect paths through WFB). Such a model showed a minimal worsening in goodness of fit indices than the previously depicted model: χ^2^ = 1737.88, df = 766, CFI = 0.95, SRMR = 0.12, RMSEA = 0.062 CI (0.058; 0.065), and NNFI = 0.95. Moreover, and above all, all the direct links from predictors to life satisfaction were not significant, providing additional and definitive evidence about the full mediation by WFB.

## 8. Discussion

The present study, grounded on Grzywacz and Carlson’s WFB theoretical model [4], examined, through a time-lagged research design, the role played by WFB on schoolteachers’ life satisfaction. In particular, its mediating role with respect to the relationship between several job (i.e., job control, coworkers support, supervisor support, and workload) and family (i.e., family support and family workload) demands and resources as antecedents, and life satisfaction as an outcome.

We contributed to the literature in different ways, in particular: (a) by examining the role played by WFB, responding to Grzywacz and Carlson’s [4] call for testing their theoretical conceptualization of WFB and to attempt to broaden and delineate its repertoire of potential antecedents. Indeed, WFB represents a construct within the context of work–family research that, to date, is still neglected when compared to other more well-known constructs such as work–family conflict and work–family enrichment; (b) by analyzing the relationship between the WFB and life satisfaction, as research on WFB’s positive outcomes has developed slowly [5,76]; and (c) by examining the role played by WFB and life satisfaction, within a specific occupational group, i.e., schoolteachers, during the COVID-19 pandemic.

The findings of this study suggest that WFB does play a significant role in the mediated relationships between family- and job-related resources, and life satisfaction.

Full and consistent support was found for some of our research hypotheses. Firstly, with regard to direct effects, higher WFB levels were more positively associated with life satisfaction, as well as with job control, supervisor support, and family support, and negatively with family workload [3,9,11].

Secondly, as regards the indirect effects of WFB between focal antecedents and life satisfaction, it was found that WFB significantly fully mediated the relationships between job control (H1a), supervisor support (H1c), and family support (H3) with life satisfaction. No significant effects were found with reference to the relationships between coworkers’ support (H1b), as well as workload (H2), and WFB.

Our results showed that job control, supervisor support, and family support can potentially improve teachers’ quality of work and family experiences, fostering a high level of WFB and thus a higher teachers’ life satisfaction. In other words, both WFB and consequent life satisfaction were found to be predicted by some specific characteristics of the job and the family domains. In general, in the wake of other studies carried out in the field of the work–family interface [8,11], our study underlines the importance that some family- and job-related resources have in the context of the work–family interface of schoolteachers. Our findings suggest that, in this context, some family and job resources play an important role, in fact, they can help schoolteachers achieve a “balance” through the accumulation of resources in a given domain; according to the COR theory [57], some resources generated in a certain domain (job and/or family) lead the schoolteachers to perceive their ability to successfully accomplish their role-related expectations in both the work and family domains.

With particular reference to the significant role played by supervisor support, it is worth discussing it in light of the relevance of personal workplace relationships [19]. Our results showed that supervisor support has the potential to promote WFB, and through this latter to improve life satisfaction. Unexpectedly, in contrast with available evidence [8], the support provided by coworkers was found insignificant. These data show that, in relation to the perception of work–family balance and life satisfaction of schoolteachers, the role of supervisors is even more important than the support provided by colleagues. Future studies should examine in more detail the dynamics underlying social support in the workplace, differentiating between different forms of support (i.e., instrumental, informative, and emotional) as well as taking into account specific personal workplace relationships (e.g., romance) that can develop within workplaces.

Moreover, results highlighted the key importance of both the work domain and the family domain in determining high levels of life satisfaction among schoolteachers. Beyond that, it should be added that the COVID-19 emergency has significantly transformed the work environment, especially for teachers, mostly women, often with a high family burden, which severely threatened their job satisfaction and quality of life [28,29].

## 9. Limitations and Future Studies

The limitations of this study primarily concern the nature of sampling. Indeed, as we recurred to a convenience sampling procedure, which does not enable us to consider the sample to be representative of the wider population, strong inferences of generalizability cannot be made.

Furthermore, this study focused exclusively on a single job category (schoolteachers); it would be worthwhile to also explore the dimensions analyzed in this research in other occupational groups. Likewise, future studies should also analyze the consequences and impact that work–family balance, life satisfaction, and teacher well-being may have on students, especially working students.

Another aspect worth mentioning concerns the design of this study; given the nature of the hypotheses of this study, and despite it being time-lagged, it is difficult to make causal inferences. Thus, future research, in order to overcome this limitation, should preferably focus more on diary studies or cross-lagged ones.

Given the importance of some determinants, other predictors should be included in future studies, to examine their effects and find out which additional job and family resources can promote WFB and consequently life satisfaction. Alternatively, it could focus on the fact that the same domains must be kept under control in order to avoid both a low level of balance between family and job domains as well as a feeling of life dissatisfaction.

In addition, the role of family characteristics, an aspect that, to date, is still neglected in the literature, should be further investigated. In fact, research into the work–family context is generally reported in a work-to-family direction and less frequently in a family-to-work direction [8]. Similarly, in future research, it would also be appropriate to analyze the role played by certain dispositional characteristics in moderating the effects of variables relating to the work–family context.

It is hereby recommended that research examines the work–family culture [77] within the school context and how this culture affects the relationship between work and family roles, as well as the relationship between the WFB and other outcomes, such as well-being, productivity, and job satisfaction [78].

## 10. Conclusions and Practical Implications

This study contributed to the work–family interface literature in several ways. In fact, despite its limitations, it might also provide significant suggestions for potential practical organizational interventions.

Firstly, research on WFB has been strengthened by establishing its relationship with a positive outcome: life satisfaction. The present study emphasizes the crucial role that WFB plays in promoting greater life satisfaction and, likewise, it points out that the WFB mediates the relationship between certain job and family characteristics and teachers’ life satisfaction.

WFB is a crucial dimension in Human Resource Management and Development practices. It is important, as it is also associated with both job and family outcomes such as job satisfaction, organizational commitment, family satisfaction, family benefits, and family functioning [11,16].

From a practical and organizational point of view, WFB could be the key to greater life satisfaction. In fact, this has important implications for organizations that should ensure they assess the WFB of their employees, as well as adequately manage certain work context-related characteristics, such as job control and supervisor support. In fact, in order to improve the perception of balance, organizations should both foster informal practices to facilitate, for example, supervisor support, and allow workers to have better autonomy and control over their tasks and work activities.

Furthermore, organizations should invest in the promotion of the WFB, by implementing adequate work–family policies [73], which can also allow workers to better manage relationships as well as manage tasks within the family environment, also in light of the importance of family support [79].

Thus, in order for this to be achieved, there must be a family-friendly work culture in all schools that makes it possible for teachers to effectively manage the tasks within the various domains of life, and school organizations should facilitate all aspects that promote an adequate balance, such as support both in the workplace and in the family [80].

The importance of supporting adequate levels of teachers’ WFB is amplified by the results achieved from these hypotheses, particularly with regard to the strong association between WFB and life satisfaction. Conversely, these results also indicate that a reduced perception of the WFB predicts lowers life satisfaction.

## Figures and Tables

**Table 1:** Study variables’ descriptive statistics and zero-order correlations.

	M (SD)	1	2	3	4	5	6	7
(1) Job control	3.68 (1.1)							
(2) Coworkers support	3.67 (0.99)	0.26 ***						
(3) Supervisor support	3.96 (1.00)	0.32 ***	0.54 ***					
(4) Workload	3.44 (0.91)	0.06	−0.11 *	−0.02				
(5) Family support	3.84 (0.78)	0.18 **	0.27 ***	0.22 ***	−0.04			
(6) Family workload	2.45 (1.31)	−0.11 *	−0.04	−0.06	−0.06	−0.26 ***		
(7) Work family balance	4.24 (0.84)	0.34 ***	0.26 ***	0.34 ***	0.03	0.26 ***	−0.15 **	
(8) Life satisfaction	4.2 (0.89)	0.24 ***	0.16 **	0.23 ***	0.03	0.18 **	−0.15 **	0.67 ***

Note: * *p* < 0.05; ** *p* < 0.01; *** *p* < 0.001.

**Table 2:** Alternative measurement models on study variables.

	χ^2^	Df	RMSEA	CFI	SRMR	NNFI
Model 1—One factor	11,272.81	819	0.22	0.84	0.28	0.38
Model 2—Eight factors	2035.01	779	0.07	0.95	0.11	0.95

## Data Availability

The data presented in this study are available on request from the corresponding author.
